# Role of Nurses in Enhancing Removable Dental Prostheses Hygiene

**DOI:** 10.1055/s-0045-1802570

**Published:** 2025-04-23

**Authors:** Ahmad Al Jaghsi, Dinesh Rokaya

**Affiliations:** 1 Clinical Sciences Department, College of Dentistry, Ajman University, Ajman, United Arab Emirates; 2 Center of Medical and Bio-Allied Health Sciences Research, Ajman University, Ajman, United Arab Emirates; 3 Department of Prosthodontics, Gerodontology, and Dental Materials, University Medicine Greifswald, Greifswald, Germany

**Keywords:** removable dental prostheses, removable partial denture, denture cleaning, dental hygiene, nurses

## Abstract

**Objective:**

Nurses contribute significantly to the management and prevention of oral diseases by educating patients on correct removable dental prostheses (RDP) cleaning techniques. This article aimed to present the role of nurses in enhancing RDP hygiene and provide a practical guide for nurses.

**Materials and Methods:**

A thorough literature search was done regarding the role of nurses in enhancing RDP hygiene and providing a practical guide for nurses in Google Scholar and PubMed from 1984 until 2024. All the English-language papers were included in this article. Publications included original research papers, review articles, and book chapters. The articles included were reviewed and added to this report.

**Results:**

Good oral hygiene is crucial in preventing infections, enhancing patient comfort, and reducing health risks like pneumonia or gum disease that can result from inadequate RDP care. Hospitals and healthcare providers are encouraged to prioritize RDP care protocols, offering training and ensuring access to necessary cleaning supplies to support nurses in their care. Nurses have an important role in supporting proper care for patients using removable dentures.

**Conclusion:**

Through best practices in RDP cleaning, correct storage, educating patients on how to care for their RDP, proper documentation, and follow-up, nurses can make a significant improvement in patient health outcomes.

## Introduction

**Fig. 1:**
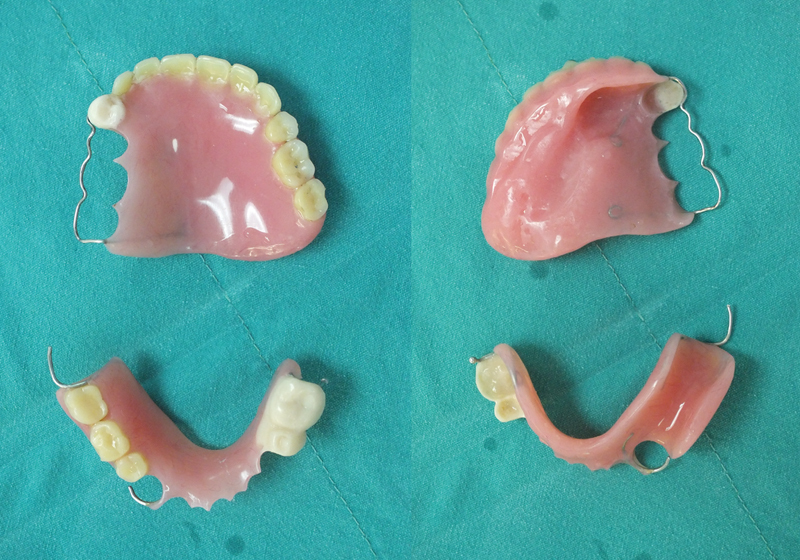
Maxillary and mandibular acrylic removable denture prosthesis (RDP).

Hospitalization provides a unique opportunity for care providers and patients to focus on and improve oral and dental hygiene, both during the hospital stay and after the patient returns home. Providing oral hygiene to hospitalized patients is an important, low-cost intervention that can enhance overall oral health. Nurses contribute significantly to the management and prevention of oral diseases by educating patients on correct removable dental prostheses (RDPs) ( Fig. 1 ) cleaning techniques. 1 2 They undertake essential RDP care tasks for hospitalized patients who cannot manage on their own due to physical or cognitive limitations. 1 2 The importance of nurses in oral hygiene management becomes increasingly critical given that research indicates only ∼40% of dentures worn by elderly individuals are properly cleaned. 3 This statistic highlights the need for comprehensive training and attentive care from nursing staff to ensure effective oral hygiene among this vulnerable group and reduce the risks of neglecting oral hygiene. Through diligent oral and RDP maintenance, nurses enhance patients' quality of life, notably improving their ability to eat, speak, and engage socially. 1 4 5

Oral bacteria can find their way into the bloodstream, increasing the risk of health complications. 6 Hence, it is essential to uphold diligent oral hygiene and ensure RDPs, complete dentures (CDs), or removable partial dentures (RPDs) are well maintained and cleaned to mitigate and manage these risks by curbing the proliferation of harmful pathogens. 6 This article aimed to present the role of nurses in enhancing RDP hygiene and provide a practical guide for nurses.

## Materials and Method

A thorough literature search was conducted regarding the role of nurses in enhancing RDP hygiene and providing a practical guide for nurses in Google Scholar and PubMed from 1984 to 2024. All the English-language articles were included in this report. Publications included original research articles, review articles, and book chapters. The included articles were reviewed and added to this report.

## Results and Discussion

### Removable Dental Prostheses (RDP) and Microbial Colonization on RDP

**Fig. 2:**
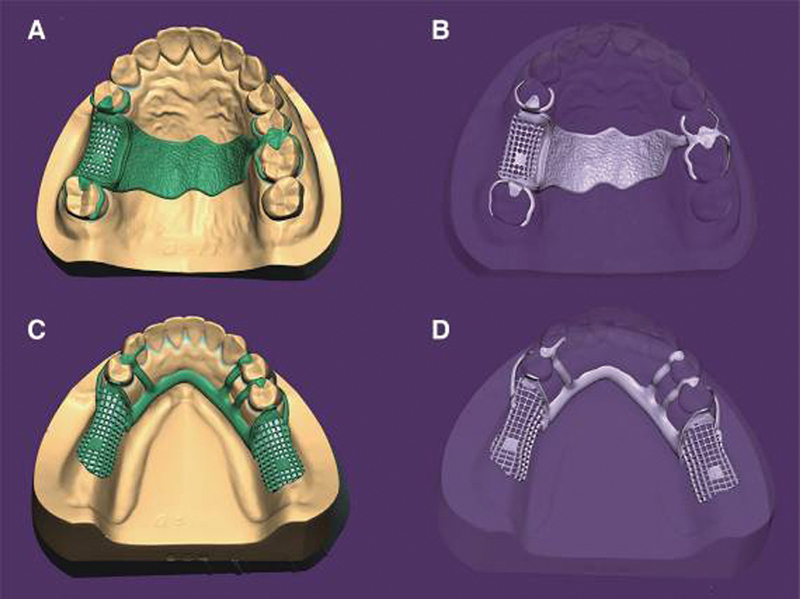
Fabrication of partial metallic removable denture prosthesis (RDP) digitally. ( **A** ) and ( **B** ) Maxillary partial RDP framework design and corresponding STL, respectively; ( **C** ) and ( **D** ) Mandibular framework design and export, respectively.

Recently, there have been various advancements in removable clinical dentistry and prosthodontics. Various new materials have been explored in the fabrication of RPD, such as poly-ether-ether-ketone, poly-ether-ketone-ketone, three-dimensional (3D)-printed metals, etc. 7 8 9 RDPs are generally fabricated from polymethylmethacrylate resins and cobalt–chromium alloys. 10 11 12 Recently, stereolithography, digital light processing, and 3D printing technologies have been used in prosthetic laboratories. 13 They can produce RDP frameworks from castable resin with designs created virtually using dental computer-aided design (CAD) software. Moreover, selective laser sintering can transfer the virtual RPD frameworks to the metallic RDP ( Fig. 2 ) with a mean accuracy of 97.452 ± 32.575 µm. 14

Furthermore, precision attachments have been used in RDPs, but studies have found that they may exert unfavorable stresses on abutments in distal extension bases. 15 RDPs of both designs showed an increase in periodontal parameters. Integrated interlock design showed better scores. It is preferable to use the attachment-retained RPD with an integrated interlock instead of a parallel interlock design.

The intaglio surface is unpolished and has a rough surface that facilitates the harboring of microorganisms. The microbial colonization on RDP is further increased after using the prostheses as a result of direct and continuous contact with the oral mucosal and salivary microbiomes. Furthermore, denture plaque and poor denture hygiene are associated with stomatitis and may contribute to oral malodor, dental caries, and periodontitis. 16 This is a great concern because RDP can also harbor pathogens that can cause respiratory infections, especially among frail older patients. 17

The accumulation of food particles, yeasts, and other microorganisms (plaque) on RDP can lead to various oral health issues, including stomatitis, papillary hyperplasia, cheilitis angularis, inflammatory fibrous hyperplasia, halitosis, dental caries, mucositis, peri-implantitis, and the accelerated deterioration of the RDP materials. 18 19 20 This kind of plaque is not just a local issue; it is also linked to systemic health problems, including diabetes and heart disease, through interactions between oral health and the body's overall wellness. 21 22 In addition, this can be a predisposing factor for *Candida* -associated denture stomatitis or compromised immunity. 23

In addition, dentures offer a reservoir for microorganisms associated with these infections, and oral bacteria have been implicated in bacterial endocarditis, aspiration pneumonia, chronic obstructive pulmonary disease, and gastrointestinal infection. It has been found that a higher rate of bacterial colonization ( *Streptococcus* species and *Klebsiella pneumonia* ) was detected on RDP after COVID-19 infection. 24 Hence, effective oral hygiene is necessary to control denture plaque biofilm and the associated oral and systemic diseases.

### Assessing RDP Cleanliness

Plaque assessment and cleaning of RDPs are done in dental clinics, hospitals, and at home to standardize hygiene evaluation and management. 25 While various methods have been proposed, no standard approach for assessing RDP plaque has been widely accepted. 25 RPD plaque assessment can be grouped into three main categories: direct visual examination, planimetric assessment, and laboratory assessment.

Laboratory assessments are essential for research and diagnosis because they provide a controlled environment for a more in-depth examination of RDP plaque. To identify and comprehend the bacteria that comprise plaque, they employ conventional microbiological methods such as swabbing, bacterial cultures, and analyses. 26 In addition, molecular techniques are employed to delve further into the genetic makeup and bacterial activities. 27 28 Through the integration of these methodologies, researchers enhance their comprehension of plaque, its influence on dental well-being, and the efficacy of distinct cleaning techniques or remedies. 27 29 In the end, these approaches contribute to better patient care and inform future research on RDP care and oral hygiene.

The planimetric assessment approach uses images of the RDP to measure the percentage of plaque-covered surfaces relative to the total surface. Advanced tools, such as image analysis software, provide quantitative data, improving precision over the subjective visual assessment. This method is primarily used in scientific research to quantify plaque accurately. 30 Plaque-disclosing dye is applied to highlight plaque, followed by taking detailed photos that are processed using software such as Image Tool 2.02, ImageJ, Photoshop, or other dental imaging tools. The software analyzes each pixel to identify plaque-covered areas. Other planimetric methods include paper-weighing quantification, point-counting (or dot-grid) methods, and various computerized approaches. 31 32 33 Additionally, some disclosing agents are fluorescent under ultraviolet light, which can be used for enhanced visualization with special lighting equipment. 33 Fluorescent dyes can be used to differentiate between live and dead bacteria within the plaque biofilm. 34 This allows for the assessment of biofilm viability and the effectiveness of cleaning procedures in reducing or eliminating live bacterial colonies. Confocal laser scanning microscopy, combined with live/dead staining, offers 3D visualization of biofilm layers on the RDP surface. 35 This technique provides detailed insights into plaque composition, thickness, and bacterial viability.

Unlike visual assessments, which can be subjective, planimetric assessment provides objective, reliable, and quantitative data, making it highly useful in research. 25 However, this method requires multiple steps, such as applying a disclosing agent, capturing images, and analyzing them, which makes it more time-consuming than the simpler visual assessments typically used in clinical settings. 25 The process demands specialized equipment such as high-quality digital cameras or scanners and software, which can be expensive. Additionally, users must be trained to handle both the equipment and software to ensure accurate plaque measurement, further increasing the complexity. 18 Due to these factors, planimetric assessment is impractical for routine clinical use and is better suited for research and specialized clinical trials where precision and detailed measurements are essential. 18 25 The applications of planimetric assessment extend across various areas of dental research. In clinical trials, it is used to measure plaque accumulation both before and after treatment, making it easier to evaluate the effectiveness of oral care products or procedures. 36 The method is also helpful in long-term studies that require tracking changes in plaque levels over time, providing clear and consistent data that can be compared across multiple points in the study. 37

In the direct visual examination, the RDPs are inspected visually for plaque accumulation. This method is particularly beneficial for monitoring patients and motivating them, as well as for checking how well they adhere to hygiene instructions. 38 Several RDP indices have been adapted from studies on natural teeth. Initially designed to measure plaque accumulation in clinical and epidemiological research, these indices form the basis for related studies. 38

**Fig. 3:**
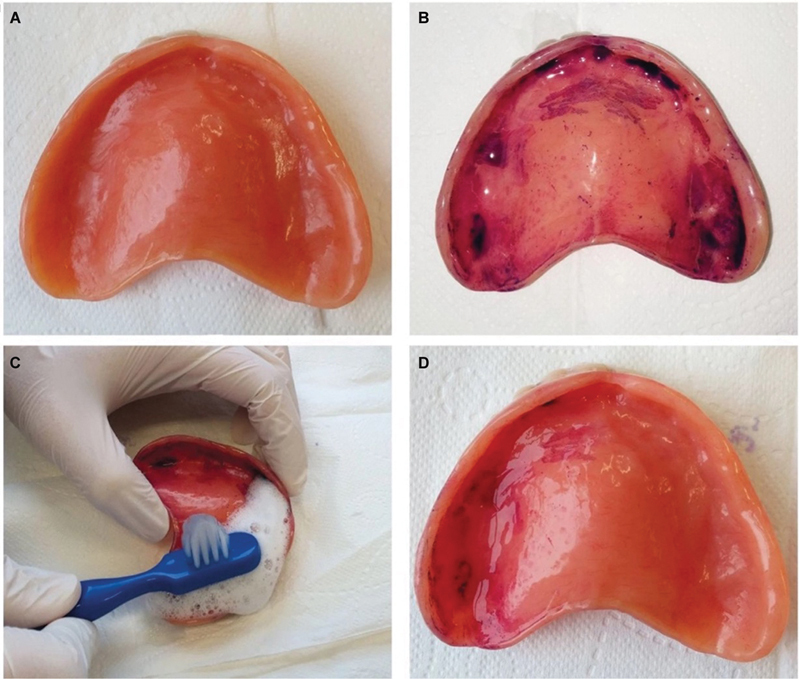
Denture cleaning index for denture hygiene instructions. ( **A** ) Denture washed gently under water; ( **B** ) application of plaque disclosing solution on tissue surface and left for 30 seconds and the denture cleanliness index score were determined; ( **C** ) denture cleaning (right tissue surface) demonstrated to the patient; ( **D** ) patient asked to clean denture (right tissue surface). (Adapted with permission from Mylonas et al. 39 )

**Fig. 4:**
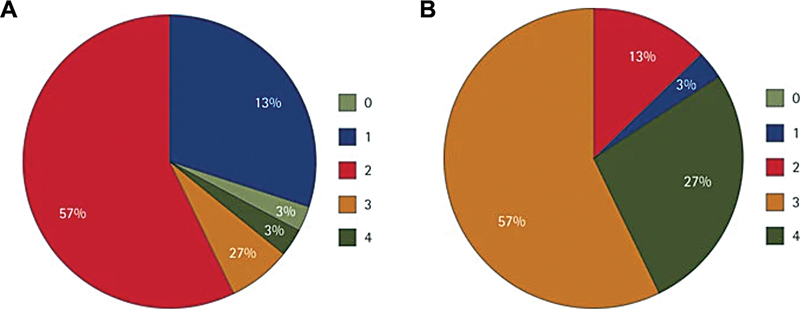
Denture cleaning index score showing an improvement in the scores before and after cleaning a combination of clinician-led patient education as well as involving the rest of the dental team and nurses. ( **A** ) Before cleaning. ( **B** ) After cleaning. Scale 0 to 4 (0 = best and 4 = worst). (Adapted with permission from Mylonas et al. 39 )

Denture cleanliness indexes (DCIs) provide a simple and rapid evaluation of denture hygiene through a grading system based on the amount of staining on the fitting surface of the denture ( Fig. 3A, 3B ). 39 DCI scores range from 0 (best) to 4 (worst) and are designated according to the DCI criteria. 40 Such assessments are favored over other assessment approaches in epidemiological research because they are quick, safe, simple, harmless, noninvasive, and low-cost methods, making them practical for widespread use. 25 41 A study by Mylonas et al 39 found that the majority of patients (84%) had poor scores (score of ≥3) initially, and through a combination of clinician-led patient education and involvement from the rest of the dental team and nurses, there was a dramatic improvement in patient DCI scores, with 90% of patients achieving denture cleanliness scores of 2 or less ( Figs. 3C, D and 4 ).

The indices implement scoring systems to quantify (score) the amount of plaque on RDP. These scores are based on predefined criteria and provide a standardized way to assess plaque. Nevertheless, indices are subjective and can vary based on the examiner's experience. 38 Plaque-disclosing agent (dye) can be used to stain the plaque, making it more visible. This helps in identifying the extent and location of plaque. Using disclosing dye and the percentage score of the plaque index appears to offer reliable information regarding the amount of plaque accumulation on RDP. 42 Erythrosine-based dyes, which are red–pink, are not only commonly used in plaque detection but are also considered among the best. 43 Additionally, two-tone disclosing agents, which stain older plaque blue and new plaque pink, are also used to help differentiate between the ages of the plaque. Based on the findings of Silva et al, the agents that exhibit the highest disclosing ability and ease of removal are 1% eosin, 1% neutral red, and 5% erythrosine. 43

Furthermore, Ambjørnsen et al 44 compared the reproducibility and reliability of different indices and reported the errors associated with denture plaque indices, noting that the internal consistency of the tested indices was good. Additionally, the indices were considered a convenient and cost-effective method for estimating plaque on RDPs. Other studies have highlighted the superiority of some indices over others. Paranhos et al 38 recommended using a computerized method, as it demonstrated better consistency and reproducibility compared with visual assessments. Additionally, they suggested avoiding certain indices and opting for specific, more reliable ones, particularly when the computerized method is not available. In addition, Paranhos et al 38 suggested that the Budtz-Jörgensen index is the preferred method for assessing plaque on CDs. Originally developed for the tissue surface of the CD, the index is straightforward and easy to use. It evaluates plaque distribution using the following scale: 0 (excellent) indicates no visible biofilm; 1 (good) means one-third or less of the fitting surface is covered with plaque; 2 (fair) represents coverage between one-third and two-thirds; and 3 (poor) shows two-thirds or more coverage. The authors propose applying this index to all types of RDP, including both complete and partial dentures, across all surfaces. They refer to this adapted version as the “modified Budtz-Jörgensen index.” Estimating the plaque through visual assessment starts with removing the RDP from the mouth. 36 Any loose food particles should be removed by rinsing the RDP under running water for 5 to 10 seconds, depending on its size. Brushing should be avoided during this process. After rinsing, the RDP should be dried with air jets for 5 to 10 seconds. A 5% erythrosine solution is spread across all surfaces of the RDP and allowed to sit for 1 minute. Afterward, the RDP is rinsed under running water for 5 to 10 seconds to wash away any unbound dye, followed by drying with air jets for 5 to 10 seconds. 36 Finally, the modified Budtz-Jörgensen index is used to estimate the plaque accumulation on the RDP in the hospital setting.

**Fig. 5:**
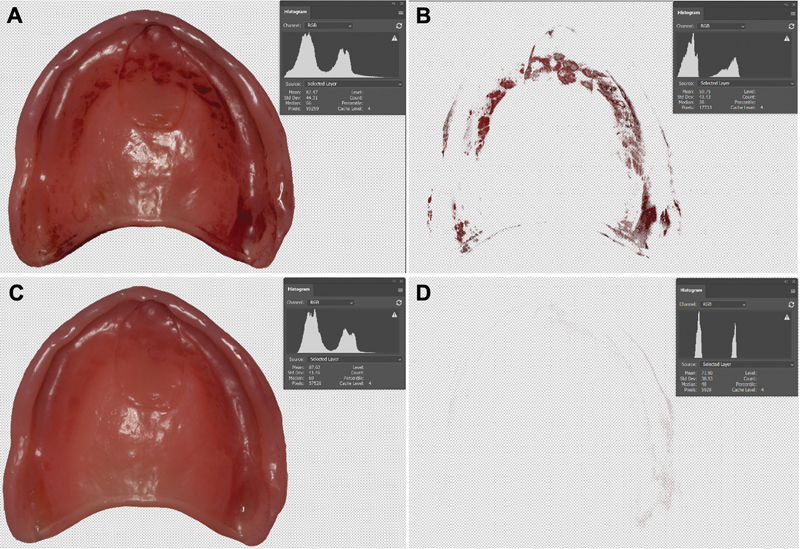
Computerized method to analyze the amount of plaque accumulation in removable dental prostheses. ( **A** ) Tissue surface of the denture with erythrosine (5%) applied; ( **B** ) plaque calculated on the Photoshop program using the white matte tool indicating plaque; ( **C** ) cleaned denture cleaned; ( **D** ) plaque calculated on the Photoshop program using the white matte tool indicating less or no plaque.

Finally, Al Jaghsi et al 18 used a new computerized planimetric method (CPM) for the measurement and assessment of the accumulation of plaque on RDPs ( Fig. 5 ). In this method, the prosthesis was rinsed under running water for 10 seconds to remove any loose food particles (without brushing). Then, it was dried with jets of air for an additional 5 seconds. Erythrosine (5%) was applied on all surfaces and left for 1 minute. Finally, the CD was rinsed under running water to remove the unbound dye for 5 seconds and dried with jets of air for 5 more seconds. Then, the denture was photographed, and using the Adobe Photoshop program, the plaque accumulation can be evaluated using the white matte tool. The CPM is suitable for clinical research because of its objectivity, reliability, high level of standardization, and ability to detect and quantify the plaque on RDP. This method can be used for patient education, accessing, and research purposes.

### Recent Approaches to RDP Hygiene Maintenance

To ensure the cleanliness and longevity of RDPs, it is essential to employ a combination of mechanical and chemical cleaning methods. 39 The mechanical procedures mainly entail brushing the RDPs with a regular toothbrush or a denture-specific brush, which reduces scratches and helps eliminate plaque and bacteria buildup. 45 Furthermore, vibratory cleaning methods such as sonic or ultrasonic baths can work effectively. Ultrasonic cleaners use high frequencies to shake loose debris from the prostheses, while sonic baths, which operate at lower frequencies, offer a more affordable but still effective alternative. 39 45

On the chemical side, several options are available to ensure thorough cleaning. Strong in getting rid of bacteria, bleach-based cleaners can be used carefully to prevent damage or discoloring of the dentures. 46 Due to their ease of use and efficiency, effervescent tablets are frequently utilized, yet over time, they could damage soft reline materials. 39 Enzyme-based cleaners are another option, targeting and breaking down organic materials on the dentures. 39 47 Mineral acid-based cleaners can effectively dissolve tough biofilm deposits but may tarnish any metal components if used improperly. 39 48 Chlorhexidine mouthwashes are also beneficial for their strong antimicrobial properties, although they can lead to staining with extended use. 49

A cleaning system known as the revolving needle device (Sympro, Renfert) combines chemical and mechanical techniques. It operates with a specialized cleaning agent called Sympro-fluid. In this process, the RDP is placed in a tub filled with Sympro-fluid. A magnet surrounding the tub activates the steel needles inside, causing them to move rapidly. As the needles repeatedly strike the denture surface, they remove plaque, ensuring more efficient cleaning compared with manual cleaning (brushing). 36 It is worth noting that several systematic reviews have underscored the importance of using both mechanical and chemical cleaning methods to maintain the hygiene and functionality of dental prostheses. This dual approach helps ensure that all aspects of denture care are covered, which minimizes the risk of infection and helps prolong the longevity of the prostheses. 39 45

**Fig. 6:**
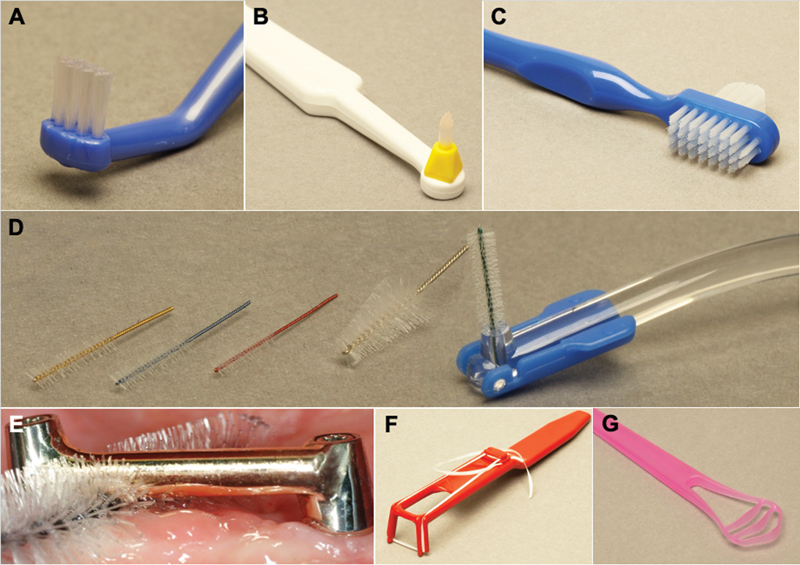
Various cleaning aids for the removable denture prosthesis, remaining teeth, and tongue cleaner. ( **A** ) Small brush; ( **B** ) interdental brush; ( **C** ) double-ended brush; ( **D** ) interdental dental brush with various sizes; ( **E** ) interdental dental brush cleaning the bar; ( **F** ) dental floss with handle; and ( **G** ) tongue cleaner.

RDP cleaning instruments and methods vary by the prostheses type. 39 Fig. 6 shows the various cleaning aids for the RDP, remaining teeth, and tongue cleaner. For polymer-based and acrylic RDPs, almost all cleaning methods are appropriate, such as denture brushes, toothbrushes, bleach-based cleaners, effervescent types, mineral-acid-based, enzyme-based cleaners, and oral rinses. However, for metal dentures, it is advisable to avoid bleach-based and mineral-acid-based cleaners to prevent potential damage. Flexible dentures should be cleaned using silicone brushes. The soft lining surfaces of RDP should not be brushed and can be cleaned using bleach-based cleaners, enzyme-based cleaners, or oral rinses. Flexible denture cleaners are also suitable for use with soft relining or any RDP type. 39 da Rosa et al 50 studied the effect of different dental prophylaxis protocols on the surface properties and their effect on the mechanical performance of CAD–computer-aided manufacturing (CAM) restorative materials. The CAD–CAM restorative materials were cleaned with the prophylactic paste fine, prophylactic paste coarse, and pumice stone, a rubber cup was attached to a handpiece operating at 2,000 rpm, and they found that the dental prophylaxis protocols do not significantly affect the mechanical strength of materials.

Maintaining RDP hygiene in hospital settings involves several challenges. Hospitalized patients, particularly those who are critically ill or have restricted mobility, rely heavily on nursing staff for RDP care. This dependency can be problematic due to staff shortages and limited time. Furthermore, many health care providers lack specialized training in RDP hygiene, which can result in poor cleaning and RDP storage practices, increasing the risk of infections such as RDP stomatitis. 19 51 Additionally, certain medications and treatments used in hospitals can reduce saliva flow, negatively impacting oral health and complicating the maintenance of RDP hygiene.

### Practical Hygiene Guides for Nurses

Nurses in a hospital setting provide crucial support to patients who are unable to care for their oral health and RDP maintenance due to various challenges. These challenges can range from physical disabilities and acute medical conditions to cognitive impairments such as dementia. Additionally, patients who are recuperating from operations, debilitated by severe fatigue, suffering from the side effects of medications, or with sensory impairments rely heavily on nurses for this essential care.

### Before Cleaning RDP

Before cleaning the RDP, the following care should be done:

- To avoid cross-contamination, the nurse should wear the appropriate personal protective equipment, gently remove the RDP from the patient's mouth, taking care not to cause discomfort, and thoroughly rinse the RDP under warm water to remove food particles and other loose debris.
- To ensure that any changes are tracked over time and to confirm with the patient or guardian the presence of RDP damage before cleaning the RDP, it is imperative that the RDP be carefully inspected to identify any damage or unusual wear. These observations should be discussed with the patient or guardian and recorded in the patient's health records.
- Apply the plaque-disclosing dye and evaluate the plaque coverage on all surfaces of the RDP following the procedure outlined earlier.
- Specify the RDP cleaning methods and list the necessary supplies and the used approach for performing the cleaning, following the approaches outlined earlier.

### RDP Cleaning

**Fig. 7:**
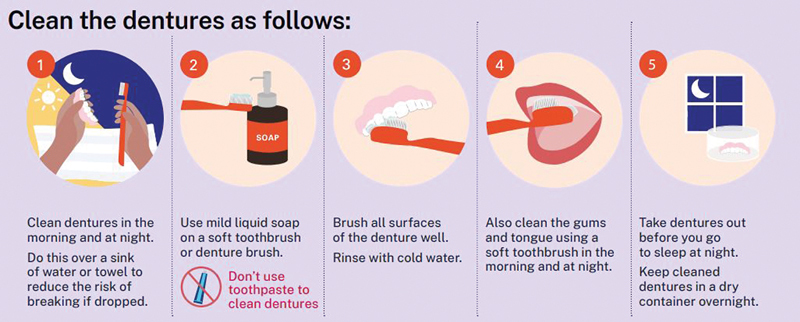
Instructions on cleaning the denture. (Reprinted with permission from Dental Health Services Victoria ©2022. 52 )

If the patient is able to clean their RDP, the following instructions should be given to the patient. Fig. 7 shows the instructions for cleaning the denture. 52 The nurse should explain the importance of maintaining good oral hygiene and encourage the patient to make RDP cleaning a daily habit. The nurse should also give the patient all the necessary cleaning supplies, such as a soft towel, an RDP container, and the recommended cleaning products chosen, to ensure proper care of the RDP. The nurse provides the patient with an orientation on intraoral hygiene, including how to clean the intraoral tissue, teeth, gums, and tongue. Additionally, the nurse instructs on the proper use of RDP cleaning supplies, ensuring that these instructions align with the company's recommendations. Moreover, the nurse emphasizes the importance of avoiding regular toothpaste and abrasive cleaners, as these can scratch the surface of the RDP, potentially causing damage. The patient is reminded to use only recommended cleaning products to maintain the integrity of the prosthesis. The nurse should explain the importance of properly storing the RDP, especially at night. To keep it from drying out or becoming deformed, the RDP should always be placed in an RDP container filled with water or a denture-soaking solution when not being worn, particularly overnight.

If the patient is unable to clean their RDP, the nurse should take responsibility for cleaning the RDP as outlined in step 5. The nurse can also use a range of tools to ensure the patient maintains optimal intraoral (teeth and soft tissue) hygiene. These include a toothbrush with warm water, an appropriate toothpaste (such as one for periodontal care, fluoride protection, or desensitizing), antiseptic mouthwash, a spit basin, and suction for patients unable to spit. A soft toothbrush can be used to clean the palate, tongue, gums, and the inside of the cheeks, along with disposable oral swabs and lip moisturizer to keep the mouth comfortable and healthy. 53

### After Cleaning RDP

The nurse encourages the patient or guardian to ask any questions or raise any concerns about their RDP care. It is crucial to explain the importance of regularly checking the RDP for signs of damage or wear and to inform the dental team if any issues arise. Additionally, the nurse reminds the patient to schedule regular dental visits with their dentist for professional cleaning and RDP maintenance. Finally, the patient is provided with a patient information leaflet for guidance and reference.

## Conclusion

By following best practices in RDP cleaning, correct storage, educating patients on how to care for their RDP, proper documentation and follow-up, nurses can make a significant difference in patient health outcomes. Hospitals and healthcare providers are encouraged to prioritize RDP care protocols, offering training and ensuring access to necessary cleaning supplies to support nurses in their care. Future research should explore ways to improve cleaning techniques and address obstacles nurses encounter, helping further improve patient care and outcomes.
